# Physical activity promotion and participation for people living with and beyond head and neck cancer: A mixed methods study

**DOI:** 10.1007/s00520-025-09198-y

**Published:** 2025-02-03

**Authors:** Hannah C. Doughty, Kerry Woolfall, Ruaraidh A. Hill, Adrian W. Midgley, Joanne M. Patterson, Lynne M. Boddy, Simon N. Rogers, Nefyn H. Williams

**Affiliations:** 1 Department of Primary Care and Mental Health, University of Liverpool, Liverpool, L69 3GL UK; 2 Faculty of Science, Liverpool John Moores University, Liverpool, L3 3AF UK; 3 Department of Public Health, Policy and Systems, University of Liverpool, Liverpool, L69 3GL UK; 4 Liverpool Reviews and Implementation Group, Department of Health Data Science, University of Liverpool, Liverpool, L69 3GL UK; 5 Department of Sport and Physical Activity, Edge Hill University, Ormskirk, L39 4QP UK; 6 Liverpool Head and Neck Centre, University of Liverpool, Liverpool, L69 3GB UK; 7 The Physical Activity Exchange, Research Institute for Sport and Exercise Sciences, Liverpool John Moores University, Liverpool, L3 2EX UK; 8 Head and Neck Centre, Wirral University Teaching Hospital, Wirral, CH49 5PE UK

**Keywords:** Barrier, Cancer, Facilitators, Healthcare professionals, Physical activity promotion, Self-efficacy

## Abstract

**Purpose:**

Head and neck cancer (HaNC) can be debilitating, resulting in high symptom burden. Physical activity (PA) can improve quality of life; however, less than 9% of HaNC patients are physically active. This study explored barriers to, and facilitators of, PA promotion and participation for HaNC patients.

**Methods:**

Semi-structured interviews with patients, family members and healthcare professionals were conducted. A questionnaire was used to measure patients’ self-reported self-efficacy (The General Self-Efficacy Scale) and patients’ and healthcare professionals’ self-reported PA (The International PA Questionnaire — Short Form). Qualitative data were analysed using reflexive thematic analysis and quantitative data were analysed descriptively. Data were synthesised drawing on the Capability-Opportunity-Motivation-Behaviour model and the Theoretical Domains Framework.

**Results:**

Twenty-eight patients, 10 family members and 18 healthcare professionals participated. Most patients self-reported moderate-to-high levels of PA and self-efficacy. Professionals self-reported high levels of PA. Patients were unaware of the benefits of PA for managing side effects and improving quality of life. Family members and professionals were fearful of patients causing themselves harm by being physically active (*reflective motivation and beliefs about consequences*). Some professionals did not consider it within their role to promote PA to HaNC patients. Many professionals stated they required training in PA promotion, and patients and family members stated they required information and guidance (*psychological capability and knowledge*).

**Conclusion:**

The responsibility of PA promotion is multidisciplinary and educating patients on the benefits and safety of PA may mitigate treatment-related side effects and improve quality of life. Future research should explore if barriers to, and facilitators of, PA behaviour change over a patient’s treatment trajectory.

**Supplementary Information:**

The online version contains supplementary material available at 10.1007/s00520-025-09198-y.

## Introduction

Head and neck cancer (HaNC) is the eighth most common cancer in the United Kingdom (UK); accounting for 3% of all new UK cancer cases [1]. HaNC can be debilitating [2], resulting in high symptom burden [3]. HaNC patients can experience dysphagia, cachexia, fatigue, pain and dyspnoea [4, 5]. Due to improvements to surgical and systemic treatments and the rise in human papillomavirus (HPV)-related HaNC, survival rates are improving [6]. However, although survival outcomes are improving, people are living longer with the long-term effects of HaNC and its treatments. Physical activity (PA) can decrease fatigue, improve body mass and functional well-being and improve quality of life for HaNC patients [7]. Despite these benefits, HaNC patients can have substantially reduced PA, and only 9% of patients met UK-recommended PA levels of at least 75 minutes of vigorous-intensity PA; 150 minutes of moderate-intensity PA; or an equivalent combination per week, and muscle-strengthening PA on two or more days a week [8].

A cross-sectional study found that although 75% of HaNC survivors were interested in participating in a PA programme, only 51% felt capable [9], highlighting that low levels of PA participation may reflect other barriers preventing patients from being physically active. The COVID-19 pandemic may have acted as a barrier to a patient’s ability to be physically active [10], and research is yet to explore the impact of the COVID-19 pandemic on PA behaviour in HaNC. Self-efficacy is an influential psychosocial determinant of PA behaviour [11] and healthcare professionals’ own levels of PA can influence their PA promotion [12]. Family members are also important determinants of a patient’s PA uptake and adherence [13]. Using behaviour change theory to understand PA promotion and participation may help to create strategies to improve promotion, uptake and adherence. Research has explored barriers to, and facilitators of, PA participation in HaNC [11, 14–17]. However, there have not been any mixed methods studies that have used behaviour change theory to explore PA promotion and participation from the perspectives of patients, family members and healthcare professionals.

The primary aim was to explore barriers to, and facilitators of, PA promotion and participation in HaNC from the perspectives of patients, family members and healthcare professionals using the Capability-Opportunity-Motivation-Behaviour (COM-B) model [18] and the Theoretical Domains Framework (TDF) [19]. Secondary aims were to (1) determine patients’ and professionals’ PA levels and explore patients’ levels of self-efficacy and (2) determine if the COVID-19 pandemic impacted PA promotion or participation.

## Methods

### Study design

A triangulation mixed methods design using the convergence model [20], including online semi-structured interviews and self-reported questionnaire data, collected and analysed in parallel with each other. Data are reported according to the Good Reporting of a Mixed Methods Study (GRAMMS) checklist [21] and the Consolidated Criteria for Reporting Qualitative Research (COREQ) checklist [22].

### Participant recruitment

This research was conducted in the North West of England between September 2021 and April 2022. Participants were recruited using a combination of purposive and snowball sampling, including National Health Service (NHS) participation identification centres (PIC) and online advertisements. Individuals identified through PICs were provided with study information by a member of the clinical team and they provided their contact details if they were interested in participating. Individuals recruited through snowball sampling or online methods were provided with study information or directly contacted the research team. Eligibility criteria are presented in Table 1. Ethical approval was granted by the Greater Manchester West NHS Research Ethics Committee (REC) (REC: 21/NW/0108; IRAS ID: 293302), and informed consent was obtained from all participants. Recruitment continued until information power [23] and sample diversity were achieved. Information power refers to the concept that the more information the sample holds relevant to the study, the lower the number of participants needed [23].

**Table 1:** Participant eligibility criteria

Patient eligibility criteria	Family member eligibility criteria	Healthcare professional eligibility criteria
• 18 years of age or older • Had a diagnosis of HaNC from any site • Were at any stage of treatment • Were recruited online, or from the National Health Service and were living in the North West of England, North Wales, or the Isle of Man • Had an Eastern Cooperative Oncology Group (ECOG) performance status grade of less than three • Had English language competence sufficient to communicate, and to comprehend and complete a questionnaire • Were willing and able to provide written informed consent	• 18 years of age or older • Were recruited online, or from the National Health Service and were living in the North West of England, North Wales, or the Isle of Man • Had English language competence sufficient to communicate • Were willing and able to provide written informed consent	• Directly involved in the care of people living with and beyond HaNC • Practising in the North West of England or North Wales • Were willing to provide written informed consent

## Materials

### Demographic and clinical characteristics

Demographic and clinical characteristic data were collected to measure sample diversity. Indices of deprivation were assessed using patients’ and family members’ postcodes of residence. The English Indices of Deprivation (IoD2019) [24] and Welsh Index of Multiple Deprivation (WIMD) (2019) [25] were used. The IoD2019 ranks deprivation deciles from most deprived (one) to least deprived (10). The WIMD ranks deprivation deciles from one (most deprived) to 1909 (least deprived). There is no comprehensive dataset that measures indices of deprivation across the Isle of Man [26].

#### GSE

The self-administrated General Self-Efficacy Scale (GSE) [27] was used to measure patients’ self-reported self-efficacy levels. The GSE is a validated 10-item self-report psychometric scale that measures optimistic self-beliefs in one’s ability to deal with demanding situations. The GSE requires individuals to rate statements on a four-point Likert scale ranging from one (not at all true) to four (exactly true). The sum of responses gives a total score that ranges between 10 and 40, with a higher score indicating higher levels of self-efficacy. The GSE produced repeatable data (Cronbach’s *α* = 0.88–0.91) and factor analysis revealed a single-factor solution, accounting for 50% of variance, relating to construct validity [28].

#### IPAQ-SF

The self-administrated International Physical Activity Questionnaire — Short Form (IPAQ-SF) [29] was used to assess patients’ and healthcare professionals’ self-reported PA levels. The IPAQ-SF is a validated population-based measure of self-reported PA amongst individuals aged 18–69 years. The IPAQ-SF consists of seven questions relating to vigorous-moderate PA, walking and sitting behaviour. PA levels were assessed using metabolic equivalent of task minutes per week (MET-min/week), and MET-min/week scores were calculated using the IPAQ-SF scoring protocol [30]. The total of vigorous, moderate and walking activities were summarised to create a total MET-min/week PA score. These scores were used to categorise individuals into one of the following categories: (1) category one: low levels of PA, (2) category two: moderate levels of PA and (3) category three: high levels of PA. The IPAQ-SF produced repeatable data (Spearman’s *p* clustered around 0.8) and criterion validity had a median *p* of approximately 0.30 [29].

### Study procedure

Semi-structured interview topic guides were researcher-derived based on previous literature, informed by the domains of the COM-B and TDF and piloted prior to use (see Online Resources 1a and 1b). Semi-structured interviews were guided by the participant and were developed iteratively after each interview. Participants were asked which remote interview method they preferred, and field notes were written after each interview. Study information was made available through the web-based survey tool Qualtrics (Qualtrics, Provo, UT). If participants were unable to access the internet, study-related material were posted and returned prior to participation. Prior to the semi-structured interview, patients were asked to answer demographic and clinical characteristic questions and the IPAQ-SF and GSE. Family members were asked to answer demographic questions and healthcare professionals were asked to answer demographic questions and the IPAQ-SF.

### Data analysis

**Fig. 1:**
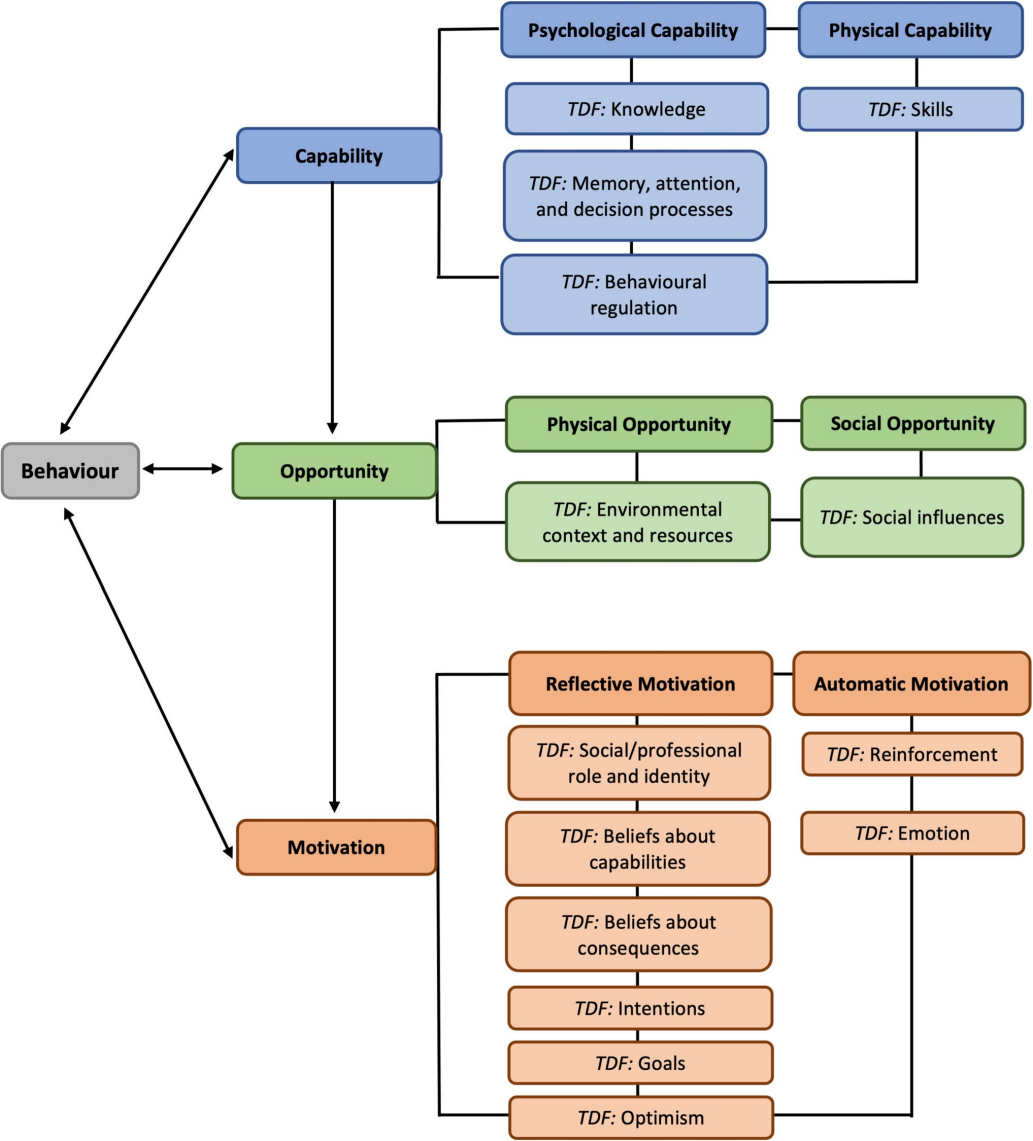
Capability-opportunity-motivation-behaviour (COM-B) [18] and theoretical domains framework (TDF) [19] behaviour change domains (adapted from [45])

Audio data were recorded digitally, transcribed verbatim by UK Transcription and checked for accuracy and anonymised by HD. NVivo 12 for MacOS (released in 2018; QSR International Pty Ltd, Burlington, MA, USA) and Microsoft Word for MacOS (Microsoft Corporation, USA) were used to develop a coding framework. Qualitative data were analysed using reflexive thematic analysis [31]. Inter-rater reliability was achieved by 10% of transcripts being independently coded. Pseudonymised illustrate quotes are presented to accompany themes. Quantitative data were collected in Qualtrics or in paper-format and were imported into Microsoft Excel and IBM SPSS Statistics for MacOS, version 28 (SPSS Inc., IBM, Chicago, IL). Normality of observed data were assessed in IBM SPSS, using standard graphical methods. GSE and IPAQ-SF data were analysed according to their scoring protocols. Patients and healthcare professionals were categorised into whether they met the Chief Medical Officers’ (CMO) PA guidelines for adults or older adults, relating to the amount of aerobic PA conducted per week [32]. Postcodes of residence were entered into the IoD2019 or WIMD online tools, which automatically generated indices of deprivation. Data were synthesised using constant comparison [33] and deductively mapped to the relevant COM-B constructs [18] and TDF domains [19]. The COM-B model posits that behaviour change is dependent upon an individual possessing the capability, opportunity and motivation in order to change their behaviour [18]. The TDF builds on the COM-B model and consists of 14 domains that aim to further understand the underlying barriers to and facilitators of evidence-based behaviour change [19] (see Fig. 1).

## Results

### Participants

**Fig. 2:**
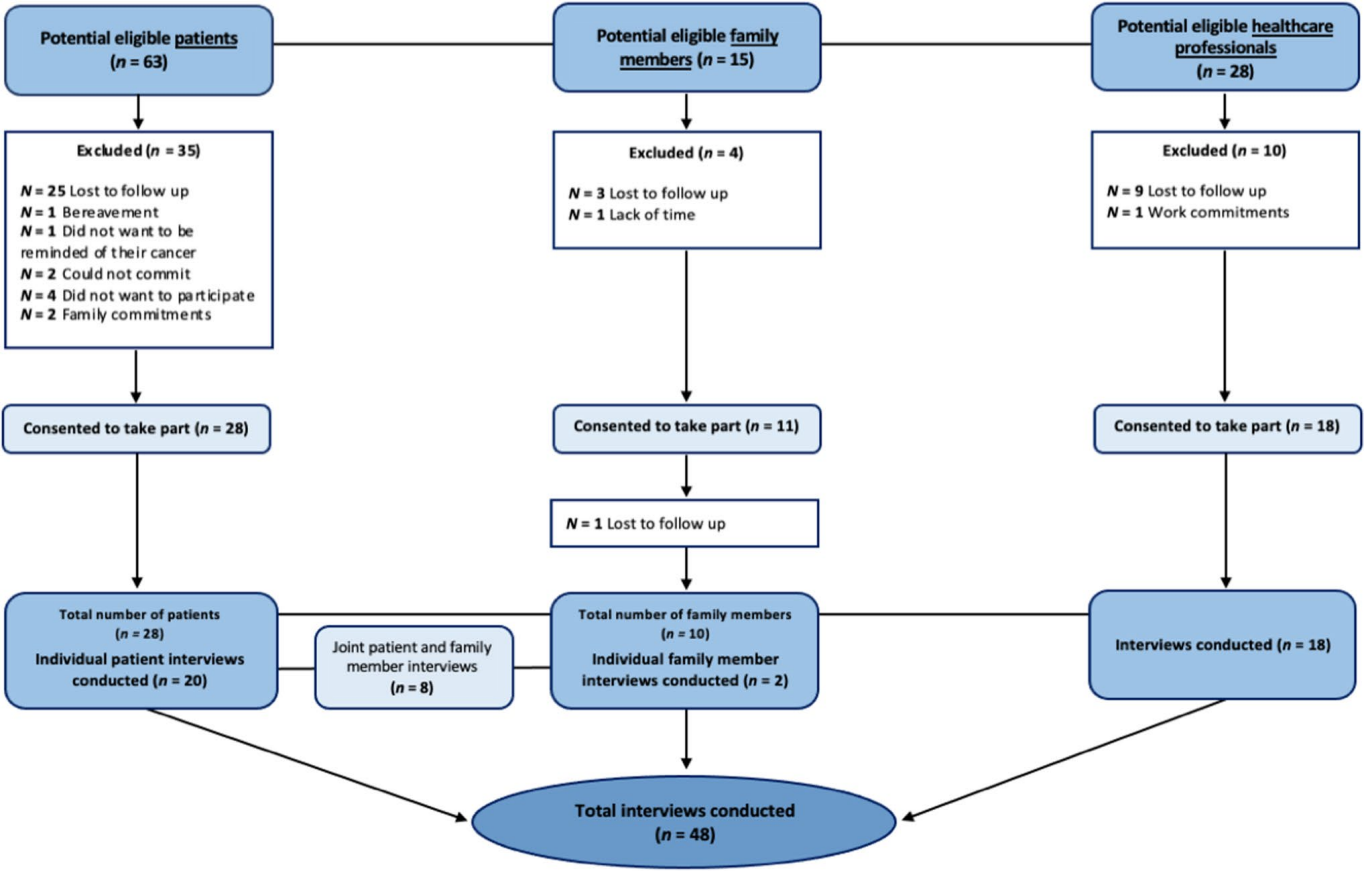
Recruitment flow diagram (*N* = 56)

Semi-structured interviews were conducted with 28 patients, 10 family members and 18 healthcare professionals (see Fig. 2). Table 2 provides an overview of participant characteristics. Patients were recruited through Liverpool University Hospitals NHS Foundation Trust (LUHFT) and had a wide range of demographic and clinical characteristics. The majority of patients were living in the North West of England (26, 93%) with deprivation deciles ranging from one (most deprived) to 10 (least deprived). Six family members (60%) were recruited through LUHFT and four (40%) were recruited through snowball sampling. The majority of family members were living in the North West of England (9, 90%), with deprivation deciles ranging from one to 10. Fifteen professionals (83%) were recruited through online advertisements, and three (17%) were recruited through snowball sampling. All professionals were practising in the North West of England and worked across a variety of healthcare settings. Interviews ranged between 16 and 110 minutes.

**Table 2:** Participant characteristics (*N* = 56)

Characteristics	Responses	*N* (%)
Patients (*N* = 28)		
Gender (patients)	Male	19 (68%)
	Female	9 (32%)
Age (patients)	Range	41–79
	Median	65
	Interquartile range	16
	Data not reported	1 (3%)
Ethnicity (patients)	White — English/Welsh/Scottish/Northern Irish/British	27 (96%)
	White — Irish	1 (4%)
Sexuality (patients)	Heterosexual	27 (96%)
	Data not reported	1 (4%)
Employment status (patients)	Employed	12 (43%)
	Retired	14 (50%)
	Self-employed	2 (7%)
Place of residence (patients)	North West of England	26 (93%)
	Isle of Man	1 (4%)
	North Wales	1 (4%)
Marital status (patients)	Married	20 (71%)
	Divorced	3 (11%)
	Single	2 (7%)
	Civil partnership	1 (4%)
	Widowed	1 (4%)
	Living with partner	1 (4%)
Education level (patients)	No qualifications	2 (7%)
	General Certificate of Secondary Education (GCSE)	10 (36%)
	Apprenticeship	3 (11%)
	A level	1 (4%)
	National Vocational Qualification (NVQ)	3 (11%)
	Undergraduate degree	3 (11%)
	Master’s degree	2 (7%)
	Doctorate	1 (4%)
	Other	1 (4%)
	Not reported	2 (7%)
Physical activity levels (patients)	Met Chief Medical Officers’ (CMO) Guidelines	22 (79%)
	Did not meet Chief Medical Officers’ (CMO) Guidelines	6 (21%)
Date of diagnosis (patients)	< 5 years ago	23 (82%)
	5–10 years ago	4 (14%)
	Not reported	1 (4%)
Treatment status (patients)	Ongoing treatment	2 (7%)
	< 5 years ago	24 (86%)
	5–10 years ago	1 (4%)
	Not reported	1 (4%)
Treatment intent (patients)	Curative	28 (100%)
Metastatic disease (patients)	No	28 (100%)
Histology (patients)	Squamous cell carcinoma	25 (86%)
	Adenocarcinoma	2 (7%)
	Neoplasm (malignant)	1 (4%)
Tumour site (patients)	Oral tongue	10 (36%)
	Floor of mouth	5 (18%)
	Mandible	4 (14%)
	Tonsil	2 (7%)
	Maxilla	2 (7%)
	Soft palate	1 (4%)
	Larynx	1 (4%)
	Right maxillary sinus	1 (4%)
	Buccal mucosa	1 (4%)
	Parotid gland	1 (4%)
Treatment received (patients)	Surgery	12 (43%)
	Surgery and radiotherapy	10 (36%)
	Chemotherapy and radiotherapy	3 (11%)
	Radiotherapy	1 (4%)
	Data not reported	2 (7%)
Comorbidities (patients)	Yes	11 (39%)
	No	16 (57%)
	Data not reported	1 (4%)
ECOG Grade (patients)	0	24 (86%)
	1	4 (14%)
Family members (*N* = 10)		
Gender (family members)	Male	3 (30%)
	Female	7 (70%)
Age (family members)	Range	25–74
	Median	56
	Interquartile range	25
Ethnicity (family members)	White — English/Welsh/Scottish/Northern Irish/British	10 (100%)
Sexuality (family members)	Heterosexual	9 (90%)
	Data not reported	1 (10%)
Employment status (family members)	Employed	5 (50%)
	Retired	4 (40%)
	Student	1 (10%)
Place of residence (family members)	North West of England	9 (90%)
	Isle of Man	1 (10%)
Relationship to patient (family members)	Wife	5 (50%)
	Husband	3 (30%)
	Daughter	2 (20%)
Professionals (*N* = 18)		
Gender (professionals)	Male	14 (78%)
	Female	4 (22%)
Age (professionals)	Range	26–57
	Median	45
	Interquartile range	19
	Data not reported	1 (6%)
Ethnicity (professionals)	White — English/Welsh/Scottish/Northern Irish/British	14 (78%)
	White — Irish	2 (11%)
	Asian/Asian British — Chinese	1 (6%)
	Other Ethnic Group	1 (6%)
Sexuality (professionals)	Heterosexual	18 (100%)
Professional role (professionals)	Nursing (Medical)	5 (28%)
	Dietetics (Allied Health)	3 (17%)
	Radiotherapy (Allied Health)	3 (17%)
	Physiotherapy (Allied Health)	2 (11%)
	Speech and Language Therapy (Allied Health)	2 (11%)
	Surgery (Medical)	2 (11%)
	General Practice (Medical)	1 (5%)
Location of practice (professionals)	North West of England	17 (94%)
	North West of England and North Wales	1 (6%)
Healthcare setting (professionals)	Secondary care	11 (61%)
	Primary and secondary care	2 (11%)
	Tertiary care	4 (22%)
	Data not reported	1 (6%)
Professional experience (professionals)	Range	5–39
	Median	18
	Interquartile range	19
Experience with head and neck cancer (professionals)	Range	2–30
	Median	10
	Interquartile range	17
Physical activity levels (professionals)	Met Chief Medical Officers’ (CMO) Guidelines	17 (94%)
	Data not reported	1 (6%)

### GSE and IPAQ-SF data

Patients self-reported a range of GSE scores, which ranged between 22 and 40 (*median* = 32, Interquartile range (*IQR*) = 9). Twelve patients (43%) self-reported moderate levels of PA (Category two, IPAQ-SF); nine patients (32%) self-reported high levels of PA (Category three, IPAQ-SF) and seven patients (25%) self-reported low levels of PA (Category one, IPAQ-SF). The majority of patients (22, 79%) met the CMOs’ PA guidelines for adults or for older adults, relating to the amount of aerobic PA conducted per week.

The majority of healthcare professionals (11, 61%) self-reported high levels of PA (Category three, IPAQ-SF), five professionals (28%) self-reported moderate levels of PA (Category two, IPAQ-SF), and one professional (6%) self-reported low levels of PA (Category one, IPAQ-SF). All professionals who reported IPAQ-SF data (17, 94%), met the CMOs’ PA guidelines for adults, relating to the amount of aerobic PA conducted per week.

Patient self-reported GSE data alongside self-reported IPAQ-SF data for both patients and healthcare professionals are presented in Table 3.

**Table 3:** Patients’ General Self-Efficacy (GSE) and International PA Questionnaire – Short Form (IPAQ-SF) Data (*N* = 28) and Professionals’ IPAQ-SF Data (*N* = 18)

Group	General Self-Efficacy (GSE) Data			International PA Questionnaire – Short Form (IPAQ-SF) Data				
	Range	Median	Interquartile range	Category 1 (low)	Category 2 (moderate)	Category 3 (high)	Median metabolic equivalent of task minutes per week (MET-min/week)	Interquartile range of metabolic equivalent of task minutes per week (MET-min/week)
				*N* (%)	*N* (%)	*N* (%)		
Patients’	22–40	32	9	7 (25%)	12 (43%)	9 (32%)	2346	3663
Healthcare Professionals’	-	-	-	1 (6%)	5 (28%)	11 (61%)	2844	3487

### Themes

Reflexive thematic analysis led to the identification of six themes. Capability-related themes included ‘lack of physical capability due to treatment-related side effects’ and ‘lack of knowledge about the benefits and importance of PA’. An opportunity-related theme was related to ‘HaNC as a disadvantaged patient cohort’ and motivation-related themes included ‘fear of causing harm by being physically active’ and ‘perceptions of who is responsible for PA promotion’**.** An additional theme related to the implications of the COVID-19 pandemic.

### Lack of physical capability due to treatment-related side effects (COM-B: physical capability; TDF: skills)

Many participants described how treatment-related side effects can create a barrier to daily functioning and PA. Professionals discussed that HaNC patients can experience treatment-related side effects that are more challenging, compared with other cancer types.

“… If you look at the treatment effects globally on a patient between head and neck radiotherapy, pelvic radiotherapy for maybe prostate or cervical cancer, and maybe breast radiotherapy… head and neck is- I think it is an accepted fact, it is way harder to get through.” (HCP4; Surgery)*.*

The most common treatment-related side effects included fatigue and difficulties in eating and drinking.

“Just tiredness… he’d only have to sit on the bed for five minutes and he’d fall asleep.” (F3).

“I was out of action for a couple of weeks. When I say action, I mean, I could not eat, I could not swallow...” (P2)*.*

### Lack of knowledge about the benefits and importance of PA (COM-B: psychological capability; TDF: knowledge)

Some patients described not understating how being physically active could help them prepare for treatment, help them day-to-day or to manage treatment-related side effects.

“If somebody said to me, “Go and walk for two miles a day” I would say, “Why? It is my neck that is bad. Walking is not going to affect my neck…” (P2).

Patients and family members described how receiving advice from healthcare professionals on the benefits and importance of PA would have been useful.

“I think just giving them a bit of advice to, it sounds a bit corny, getting a bit more active. It’s only going to benefit you later on...” (P18).

Notably, a patient described thinking their healthcare professionals did not perceive they would benefit from being physically active.

“I think maybe they perhaps don't simply register the fact that people would benefit from trying to be active.” (P17).

Professionals’ own perceptions and interest in PA influenced their discussions with patients. Those who were physically active discussed being unable to “leave that at the door when I go into my clinic” (HCP4; Surgery). While another described that if professionals are not physically active or interested in PA themselves, it is “very hard for them to encourage others to do it when it’s not what they actually do themselves…” (HCP12; Physiotherapy).

Professionals described that receiving training on the benefits of PA for HaNC would be useful. This training could include a “small module on mandatory training” (HCP17; Radiotherapy), and “more education around where to signpost” (HCP13; Nursing).

### HaNC as a disadvantaged patient cohort (COM-B: physical and social opportunity; TDF: environmental context and resources and social influences)

One patient described how she had been diagnosed with breast cancer and was offered a Macmillan-led PA class for people with breast cancer, which was “one of the best things” she had done as it helped her “physically and mentally”*.* She described how she did not understand “why they can't do that sort of thing for HaNC” (P25).

Professionals discussed that PA promotion needed to be prioritised across health services and recognised as a core factor in the treatment and recovery for HaNC.

“For me, it’s absolutely out there, exercise is medicine. It’s just not seen like that at all. But it sits in that same category. It’s all treatment on the same scale.” (HCP12; Physiotherapy)

However, a challenge to PA promotion was to “look at what are you prioritising it over” (HCP5; Speech and Language Therapy).

### Fear of causing harm by being physically active (COM-B: automatic motivation and reflective motivation; TDF: emotion and beliefs about consequences)

Many participants expressed fear of overexertion, and lacked confidence in what they were able to do and were fearful of weight loss. One family member described that she thought if her father lost weight, it meant he was at risk of having a recurrence.

“He had had an appointment and (a doctor) had said that he had never had a patient where they had gained weight and they had a recurrence. So, it is sort of like…in my head, I just thought, “Well, if he loses weight, what does that mean?” … he is more at risk of having a recurrence.” (F4).

One patient described being advised to do less PA by their healthcare professional and feeling worse as a result.

“When I found out what it was, she was wanting me to do compared to what I had been doing, I thought “Oh God, I’m going to rot in my chair.”… I gave it a go anyway and I must admit I didn’t feel as good as what I had done.” (P26)

Several professionals discussed that if patients burnt too many calories and lost weight, this could have had a detrimental impact on their treatment.

“… We don’t want them to lose weight, and then we explain the reason, because we don’t want the mask to be gappy. So, the consequence if the immobilisation doesn’t fit to the patient, is that they have to go through the re-scan and re-plan stage, which makes their treatment longer.” (HCP7; Radiotherapy)

### Perceptions of who is responsible for PA promotion (COM-B: reflective motivation and physical opportunity; TDF: social/professional role and identity and environmental context and resources)

Many professionals described they did not see it as part of their role to discuss PA with their patients.

“I have not had a great deal of discussions about PA with patients, because I do not have to.” (HCP6; Nursing)

The majority of professionals discussed how PA promotion needed to be a multidisciplinary approach to ensure that a consistent message was being conveyed to patients. However, one professional identified that a consultant’s input was imperative when encouraging and facilitating behaviour change.

“There’s so much anecdotal evidence out there that the patients do everything that their consultant says, so if it’s coming from them, it’s more embedded...” (HCP1; Physiotherapy)

One professional described how other health promoting behaviours were discussed in consultations, as these factors are involved in the *“*aetiology of the disease and the response to treatment” (HCP4; Surgery).

“We have got as far as smoking, alcohol, generally a healthier lifestyle, diet [advice]… and there are a couple of good reasons for that… It’s more medicalised anyway, it’s part of your medical history, more typically… and it can be just a straight limitation about how you manage the patients pre-operatively.” (HCP4; Surgery)

### Implications of the COVID-19 pandemic (COM-B: physical capability and physical opportunity; TDF: skills and environmental context and resources)

Some patients discussed being less physically active during the pandemic and some professionals discussed that as a result of lower PA levels, patients presented with reduced *physical capability* prior to treatment.

“After COVID and everything, that has just thrown everything out the window because we've stopped for a year and a half…” (P4)

“A lot of people now, not so much at the start of the pandemic but now, are saying, “I’ve not done anything for the past 18 months because of COVID…” So, a lot of patients are coming through to have their surgery saying, “I’m deconditioned anyway.” (HCP1; Physiotherapy)

One professional discussed that as patients with cancer were advised to self-isolate during the COVID-19 pandemic, when this advice was lifted, they forgot to promote PA again with their patients.

“I wasn’t really discussing it with patients so much… when patients could actually start going out and things again, I had forgotten to mention it…” (HCP11; Radiotherapy)

Notably, one professional discussed the results of a surgical research trial conducted during the COVID-19 pandemic, which found that self-isolation was detrimental to patients.

“It said, ‘isolation before elective surgery might be associated with a small but clinically important increased risk of post-operative pulmonary complications.’… It just suggested that we might be doing a harmful thing for our patients by telling them to go and hide away in the cupboard because COVID is out there.” (HCP2; Surgery)

## Discussion

### Summary of main findings

These findings indicate that treatment-related side effects can significantly impact a patient’s *physical capability*, resulting in a barrier to PA participation. Many patients were unaware of the benefits of PA for mitigating and managing treatment-related side effects and improving quality of life. Family members and professionals were fearful of patients causing themselves harm by being physically active, indicating that *psychological capability* and *automatic* and *reflective motivation* were key barriers. PA was not consistently promoted to patients, and this may be due to the lack of *physical opportunity* available for people living with and beyond HaNC to participate in PA programmes. However, it could also be explained by *reflective motivation*, as some professionals did not consider it as part of their role to promote PA. The COVID-19 pandemic had a detrimental impact on patients’ PA levels, with professionals describing that patients presented with reduced physical abilities, prior to treatment. *Psychological capability* featured as a key facilitator to PA promotion and participation, as many professionals expressed a need for training in PA promotion, and patients and family members discussed requiring information, support and guidance.

### Comparison with previous literature

Despite the majority of patients self-reporting moderate-to-high levels of PA, the qualitative findings indicated that a patient’s actual PA behaviour may be lower than self-reported scores. The self-reported IPAQ-SF scores in the current study indicated that only 21% of patients did not meet the CMOs’ PA guidelines for adults or older adults, relating to the amount of aerobic PA conducted per week. This finding contradicts previous research conducted in the UK, who found that as many as 66% of HaNC patients were insufficiently active to gain appreciable health benefits [34]. This may be explained by the previous study using the Godin Leisure-Time PA Questionnaire (QLTPAQ), while the present study used the IPAQ-SF. The GSLTPAQ requires individuals to define the duration of each activity during a typical seven-day week, whereas the IPAQ-SF asks individuals to define and quantify their activity levels during the last seven days. This could also be explained by self-reported PA data being subject to bias, and may not be a true indication of an individual’s PA levels [35]. Despite this, the qualitative findings identified barriers to PA participation. Many patients were fearful of being physically active in case they lost weight and their treatment was impacted. Similarly, a family member expressed concerns that weight loss meant recurrence of disease. This concern was echoed in a study conducted with breast cancer survivors, who found that family and friends confused weight loss from PA, with the progression of cancer [36]. Although the prevalence of weight loss in HaNC has ranged between 31% and 57% during-treatment [37]; 77% of weight loss has been shown to be attributable to loss of lean body mass [38]. PA, particularly resistance training, can support weight gain by mitigating skeletal muscle atrophy commonly associated with cancer and its treatment, as well as counteracting the effects of physical inactivity [39].

Patients described lacking knowledge regarding how being physically active could have helped them prepare for treatment or to manage any treatment-related side effects. A study conducted with people with colorectal cancer identified that patients could lack knowledge regarding the importance of PA for disease management [40]. Despite the majority of self-reported GSE data indicating that patients had moderate-to-high levels of self-efficacy, patients expressed concerns over fear of overexertion and lacked confidence in their own abilities to be active. This finding was consistent with a study conducted with people with breast, prostate and colorectal cancer who found that patients had reservations about their own ability to be physically active [41]. However, previous research has suggested the benefits of PA have been shown to outweigh any potential risks for people with long-term conditions [42]. The current study identified that the COVID-19 pandemic had a detrimental impact on patients’ PA levels. Previous research has found pre-operative isolation was associated with a 20% increased risk of post-operative pulmonary complications, and this finding was consistent after being adjusted for age, comorbidities and type of surgery performed [43]. These findings indicate that self-isolation may result in patients reducing their levels of PA, which conversely led to functional decline and adversely influenced post-operative outcomes [43].

### Strengths and limitations

This was the first mixed methods study to use the COM-B and TDF to explore patients’, family members’ and professionals’ views and experiences of PA for HaNC. Using purposive sampling enabled a variety of demographic, clinical characteristics and perspectives to be collected. Limitations include the IPAQ-SF not including questions related to resistance or flexibility training and self-reported data may not be a true reflection of an individual’s PA levels. Despite the broad eligibility criteria, the HPV status of patients were not collected. HPV-positive HaNC patients are more likely to be asymptomatic [44], and this subgroup of patients may report less barriers to PA participation. As HPV status was not collected, the current sample may not reflect the difference in complexities in HPV-positive and HPV-negative HaNC.

### Implications for practice and future research

Many patients lacked the knowledge and motivation to become, or to continue being PA, with fear of harm being detrimental to PA promotion and participation. Behaviour change techniques that focus on improving *psychological capability* and *reflective motivation* by enhancing knowledge regarding the benefits and importance of PA may ensure patients are less fearful of being physically active. Second, providing professionals with training in PA promotion, including where to signpost patients for further information, may ensure the importance of PA is enforced and reinforced to patients. Lastly, as people with HaNC are living longer after treatment, future research is needed to explore the individualised needs of patients and to consider how the barriers to, and facilitators of, PA behaviour may change over a patient’s treatment trajectory.

## Conclusion

This study drew upon the COM-B and TDF behaviour change theoretical domains, to help understand barriers to, and facilitators of, PA promotion and participation, for HaNC patients. Many patients expressed fear of causing themselves harm by being physically active and lacked knowledge regarding how being physically active could help them prepare for treatment or to manage any treatment-related side effects. Findings suggest that PA promotion should be a multidisciplinary approach and providing professionals with training may help to ensure the importance of PA is consistently enforced and reinforced to patients. Despite the majority of patients self-reporting moderate-to-high levels of PA, the qualitative findings indicated that a patient’s actual PA behaviour may be lower than self-reported scores. Future research should explore how the barriers to, and facilitators of, PA behaviour may change over a patient’s treatment trajectory, and future PA interventions should be developed using the current theory-based findings.

## Supplementary Information

Below is the link to the electronic supplementary material.

Supplementary file1 (DOCX 31.7 KB)

## Data Availability

The datasets generated are predominately qualitative and are available from the corresponding author on reasonable request.
