# Supplementary material for: Physical activity promotion and participation for people living with and beyond head and neck cancer: A mixed methods study
Source: Support Care Cancer. 2025 Feb 3;33(2):141. doi: 10.1007/s00520-025-09198-y (PMC11788227; doi:10.1007/s00520-025-09198-y)
Supplement: Supplementary file 1 — Supplementary file1 (DOCX 31.7 KB) [file 520_2025_9198_MOESM1_ESM.docx]

**Online Resource 1a.** Patient and Family Member Capability-Opportunity-Motivation-Behaviour (COM-B) and Theoretical Domains Framework (TDF) Informed Interview Schedule

| **COM-B Main Construct** | **COM-B Smaller Construct** | **TDF Domain(s)** | **Question** |
| --- | --- | --- | --- |
| **Capability** | **Physical capability** | **Skills** | What physical activities do you currently do? *(Both)* |
|  |  |  | Have your activity levels changed from pre to post diagnosis? (Can you please elaborate?) *(Patients)* |
|  |  |  | Can you talk about whether there are any types of physical activities you think you would like to do but can’t? *(Patients)* |
|  | **Psychological capability** | **Knowledge** | Have you discussed physical activity with any of your healthcare professionals? *Prompt: If so, who have you discussed this with? (Both)* |
|  |  |  | Do you recall receiving information about physical activity from any of your healthcare professionals since the diagnosis? *Prompt: If so, what has been discussed with you? (Both)* |
|  |  |  | Can you talk about whether you think it would have helped to have received information about physical activity? *Prompt: What type of information would you have liked? (Both)* |
|  |  |  | When do you feel would be the best time for healthcare professionals to discuss physical activity? *(Both)* |
|  |  |  | How do you think that being physically active might impact on dealing with cancer and the treatment received? *(Both)* |
|  |  | **Social/professional role and identity** | Would you prefer to receive information about physical activity from a particular healthcare professional? *(Both)* |
| **Opportunity** | **Physical opportunity** | **Environmental context, and resources** | *(If information provided)* What information have you received about physical activity from your healthcare professionals since the cancer diagnosis, and what forms of physical activity have been offered *(e.g., enrolment in group supervised physical activity classes, leaflets, being encouraged to be more active in general)*? *(Both)* |
|  |  |  | Do you have facilities available where you live where you can be physically active? Or do you live in an area where it is easy to be physically active? *(Both)* |
|  | **Social opportunity** | **Social influences** | Do you have family members/friends who enjoy being physically active? *(Patients)* |
| **Motivation** | **Automatic motivation** | **Emotion** | What are your thoughts about physical activity in general? Prompt: Is it something that you enjoy or don’t enjoy doing, do you think it benefits you in any way? (Both) |
|  | **Reflective motivation** | **Beliefs about capabilities** | Can you talk to me a little about whether you would describe yourself as an active person? *(Both)* |
|  |  | **Beliefs about consequences** | Do you have any concerns about being active? *(Both)* |
|  |  |  | Can you talk about how you think these concerns could be managed? *(Both)* |
|  |  | **Goals**  **Optimism** | Would you like to become more active? If yes: What do you think will help you become more active if this is something that you are wanting to do? (Both) |
|  |  | **Intentions**  **Reinforcement** | Can you talk about what motivates you to be physically active? Or what would motivate you to be active? *(Both)* |
| **Questions related to all COM-B constructs and TDF domains** | | | What are some of the main things that help you to stay physically active? *(Both)* |
|  |  |  | Is there anything that prevents you from being active? What are the main factors that prevent you from being physically active? *(Both)* |
|  |  |  | Have you experienced any challenges that have made it difficult for you to be physically active? *Can you tell me a little bit about what these challenges were? (Both)* |
|  |  |  | Could you talk about how you would manage these challenges/ how you feel that activities could be tailored to help you manage these challenges? *(Both)* |
|  |  |  | Do you feel that you need support in order to become physically active? *What support do you think you need/ would benefit from? (Patients)* |
| **Questions related to the impact of COVID-19** | | | Can you talk about if and how the coronavirus has impacted you? |
|  |  |  | Has anything about the coronavirus impacted your ability to be physically active *(for example, lockdowns, concerns over the virus itself)*? *(Both)* |

**Online Resource 1b.** Healthcare Professional Capability-Opportunity-Motivation-Behaviour (COM-B) and Theoretical Domains Framework (TDF) Informed Interview Schedule

| **COM-B Main Construct** | **COM-B Smaller Construct** | **TDF Domain(s)** | **Question** |
| --- | --- | --- | --- |
| **Capability** | **Psychological capability** | **Knowledge** | In your opinion, how important do you think it is for patients to become or continue to become physically active? |
|  |  |  | Are you familiar with any guidelines to provide to people living with and beyond head and neck cancer about physical activity? |
|  |  |  | When do you think information on physical activity should be provided to people living with and beyond head and neck cancer? |
|  |  |  | Have you received any formal training either through your degree or speciality training on how to deliver advice about physical activity? (Can be general or cancer specific) *Prompt:* *Was this general or cancer specific?* |
|  |  |  | Do you think you would benefit from some training in this area? If so, what type of training do you think would be useful? |
| **Opportunity** | **Physical opportunity** | **Environmental context, and resources** | Are you aware of any services or resources available at your hospital, or in your local area to refer people living with and beyond head and neck cancer for physical activity advice or support? |
| **Motivation** | **Automatic motivation** | **Social/professional role, and identity** | Do you see it as part of your role to discuss physical activity with people living with and beyond head and neck cancer? *Prompt: If not, why do you feel that it is not part of your role?* |
|  |  |  | Do your patients discuss physical activity with you? |
|  | **Reflective motivation** | **Beliefs about capabilities** | Do you feel confident raising discussions around physical activity with people living with and beyond head and neck cancer? |
|  |  | **Emotion** | Do you think the promotion of physical activity is prioritised, or do you think it needs to be prioritised? *Prompt: Could you talk a little bit about why this is?* |
| **Questions related to all COM-B constructs and TDF domains** | | | What in your opinion are patients’ attitudes towards physical activity in general? |
|  |  |  | In your experience, how can the symptoms that people living with and beyond head and neck cancer can experience, impact their ability to be physically active? |
|  |  |  | How do you think that these challenges could be addressed to help people living with and beyond head and neck cancer to become/stay active? |
|  |  |  | Would you benefit from anything in particular in order to help you discuss physical activity with people living with and beyond head and neck cancer? |
|  |  |  | Can you talk about what you think might motivate people living with and beyond head and neck cancer to be active? |
|  |  |  | How do you think physical activity could be promoted better within the health service? |
|  |  |  | How do you think we could help people living with and beyond head and neck cancer to be more active? What sorts of things do you think would be helpful? |
| **Questions related to the impact of COVID-19** | | | Can you talk a little bit about whether the coronavirus has had, or is having an impact on the patients living with and beyond head and neck cancer you, see? |
|  |  |  | If and how has the coronavirus impacted your discussions about physical activity with patients? |
